# Treatment of upper tract urothelial carcinoma with ureteroscopy and thulium laser: a retrospective single center study

**DOI:** 10.1186/s12885-018-4118-y

**Published:** 2018-02-17

**Authors:** Jin Wen, Zhi G. Ji, Han Z. Li

**Affiliations:** 1 Department of Urology, Peking Union Medical College Hospital, Beijing, China; 2 Department of Urology, Peking Union Medical College Hospital, Chinese Academy of Medical Sciences & Peking Union Medical College, Shuai Fu Yuan 1, Wang Fu Jing Street, Beijing, 100730 China

**Keywords:** Laser therapy, Upper tract, Urothelial carcinoma, Ureteroscopic treatment

## Abstract

**Background:**

Treatment with the combination of ureteroscopy and thulium laser ablation may provide an alternative to radical nephroureterectomy (RNU) for patients with upper tract urothelial carcinoma (UTUC). The purpose of this study was to investigate the efficacy and safety of this technique.

**Methods:**

We performed a retrospective review of the data for patients who were treated surgically for upper tract urothelial carcinoma in a single center. It included 32 patients treated by endoscopic thulium laser resection and 107 patients treated by radical nephroureterectomy (RNU). We compared the data of patient sex, age at diagnosis, location of carcinoma, length of hospitalization, tumor site, size, grade, recurrence, preoperative creatinine and postoperative creatinine in two groups. Patients were examined by ureteroscopy every 3 months during the first year after surgery, then every 6 months each year.

**Results:**

All 32 patients were treated successfully, among which 6 were operated by a flexible ureteroscope. The average tumor size was 13 ± 7 mm in diameter. The tumor was rated as low grade in 27 patients and high grade in 5 patients. Ureteral stricture developed in 4 patients 3 months later after surgery, but the stricture was succesfully treated through endoscopic dilation. Seven patients had tumor recurrence, 3 of which underwent nephroureterectomy during the follow-up. Postoperative creatinine levels (umol/L) were respectively 89 ± 7.5 in laser group and 123 ± 15.4 in RNU group (*p* < 0.01). Length of hospitalization was respectively 3.6 ± 1.9 and 8.6 ± 2.4 days (*p* < 0.01). Local or bladder recurrence rate of thulium laser group and RNU group was respectively 21.9 and 13.1% (*p* < 0.01).

**Conclusions:**

Thulium laser group is associated with a less loss of renal function, a shorter length of hospitalization, but a higher rate of tumor recurrence. Thulium laser therapy combined with ureteroscopic treatment can be considered as an acceptable treatment for selected cases of UTUC. Lifetime intensive surveillance is necessary.

## Background

Upper tract urothelial carcinomas (UTUC) are rare, which accounts for only 2 to 5% of urothelial tumors. Nephroureterectomy with bladder-cuff removal is still the standard treatment for UTUC [1]. However, with the development of technology, such as endoscopes and laser ablation, urologists can approach the upper urinary tract and resect tumor easily [2]. Over the last decade, Thulium laser ablation has been proven to be a promising management to treat benign prostatic hyperplasia (BPH) and bladder tumors when compared with holmium laser or neodymium laser [3, 4]. However, the use of thulium laser for treatment of UTUC, which may also have a great clinical importance, is rarely reported. Nephron sparing endoscopic approach may be preferable in selected patients especially those with low-grade tumor, solitary kidney, renal insufficiency or bilateral UTUC [5]. Some of the patients who have small localized tumors and with a normal contralateral kidney can also be treated by endoscopic approach [6]. We report 32 cases of upper tract urothelial carcinomas treated with 1.9 μm thulium laser to evaluate the use of this technique for UTUC.

## Methods

After obtaining the ethics committee opproval at our institution, we reviewed 32 patients treated with 1.9 μm thulium laser via ureteroscope for upper tract urothelial carcinomas from January 2013 to January 2017. Patients were well informed about the risks and benefits of simple endoscopic thulium laser treatment (ETLT). Surgeries were performed by several experienced urologists. All patients were placed in routine lithotomy position. The instruments used for surgery included 8–9.8F rigid ureteroscope (Wolf, Germany), flexible ureteroscope (Olympus, Japan), ureteral access sheath (Cook, USA), and Vela® XL 1.9 μm laser system (Starmedtec, Germany). The delivery system was a bare-ended laser fiber with an optical core diameter of 200-600 μm and its energy was 30-50 W. A black hydrophilic guidewire was placed in the renal pelvis under rigid ureteroscope. All the patients were diagnosed endoscopically. Specimens were obtained through a cup forcep and were confirmed by biopsy. A 5-Fr ureteral “D-J” stent was left in situ at the end of the procedure, which could prevent transient ureteral edema and ensure postoperative drainage. The stent was usually removed 3 months later for decreasing the rate of ureteral stricture. All samples were sent to the laboratory and were assessed by a single specialist. Patients were followed with ureteroscopy at 3-month intervals during the first year and 6-month intervals since the second year. Tumor recurrence was defined as visible tumor either in ureter or bladder. Laparoscopic nephroureterectomy was indicated in some patients with high-grade or progressive disease. Continuous variables were shown as mean ± standard deviation and categorical variables as numbers and proportions. Group differences were assessed with t tests and Chi-square tests where appropriate. The software SPSS statistics 18.0 was used for all analyses.

## Results

All 32 patients were treated successfully, 6 of which were managed with a flexible ureteroscope. The patients were aged 69.3 ± 11 years at the time of diagnosis. The average tumor size was 13 ± 7 mm in diameter. There were 13 right-sided tumors and 19 left-sided tumors. Tumors were located in the renal pelvis in 4 patients, in the ureter in 28 patients. There were no major perioperative complications such as ureteral perforation or bleeding necessitating transfusion. None of the patients required open surgery and blood transfusion. The tumor was determined as low grade in 27 patients and high grade in 5 patients. Four patients developed ureteral stricture 3 months after operation, which was treated by stenting, balloon dilatation, or laser incision. Data of the Laser group were compared with 107 consecutive RNU cases from January 2013 to January 2017, which were operated by open bladder cuff in 83, endoscopic bladder cuff resection in 24. Results were shown in Table 1. Tumor recurrence rate of laser group was higher than RNU group, which was 21.9 and 7.8% (*P* < 0.01), respectively. Length of hospitalization was significantly shorter in laser group (*P* < 0.01), which was 3.6 ± 1.9 days, compared with 8.6 ± 2.4 days in RNU group. Postoperative creatinine level (umol/L) in laser group was lower than in RNU group, which was 89 ± 7.5 and 123 ± 9.4 (*P* < 0.01), respectively. Seven patients in laser group had tumor recurrence, 3 of which required nephroureterectomy due to high-grade and progressive disease. No metastatic disease was found during the follow-up.

**Table 1:** Item of data in Laser group and RNU group

Item	Laser	RNU	*P* value
Number of patients	32	107	–
Male/female	21/11	76/31	> 0.01
Right/left	13/19	41/66	> 0.01
Age (years)	69.3 ± 11	62.3 ± 9.4	> 0.01
Length of hospitalization	3.6 ± 1.9	8.6 ± 2.4	< 0.01
Renal pelvis/ureter	4/28	29/78	< 0.05
Low / High grade	27/5	75 /32	< 0.01
Tumor size (mm)	3 ± 7	23 ± 15	< 0.01
Cr level 1 day post-operatively(umol/L)	89 ± 7.5	123 ± 9.4	< 0.01
Recurrence rate	7/32 (21.9%)	8/107 (7.8%)	< 0.01

## Discussion

We presented our experience of endoscopic thulium laser resection for UTUC. UTUC is commonly more invasive and lethal than bladder cancer [7]. Traditional nephroureterectomy may leave patients functionally anephric and lead to a high morbidity and mortality associated with dialysis. Data from end-stage renal disease database showed a decrease in survival in older patients. In a recent study which comprised 128 hemodialysis patients with a mean age of 61, the 3-year survival was only 55%. Ablative therapies, such as electrocoagulation and laser ablation, were considered as minimally invasive alternatives to nephroureterectomy over the past years. Different lasers, especially holmium and neodymium lasers, have been proven to be useful for complete resections of small, superficial and low-grade tumors. Thulium laser system was investigated over the last decade, which also had an advantage in the management of urological disease. It provides a continuous wave which can generate sharply defined incisions and obtain excellent coagulation, rather than a pulsed laser [8]. In a study, thulium laser was reported to deliver recurrence-free survival superior to holmium laser ablation and reduced bleeding and mucosal perforations [9]. Owing to the rapid development of new technologies (flexible, ureteral sheaths) and a vast experience of endoscopic treatment, endoscopic thulium laser resection of UTUC has gained popularity. The technique is emerged out of a need for renal preservation in patients with solitary kidneys, renal insufficiency, bilateral tumors, or a high risk of complications associated with surgery. Endoscopic thulium laser resection of UTUC has stimulated widespread use of nephron-sparing surgery, which does not compromise oncologic outcome and renal function and can reach both the renal pelvis and the ureter [10, 11]. In our study, the Vela® XL 1.9 μm thulium laser system with wavelength of 1920 μm was used. The 1.9um thulium laser can be delivered with flexible fibers. The depth of thermal injury caused by this laser is only about 0.1 mm, which can be used to achieve precise, relatively scar-free surgery to the surface of the ureter [12]. We used the zebra guide wire during incision to avoid formation of an iatrogenic false channel. The benefits of this minimally invasive technique included a less loss of renal function and a shorter length of hospitalization (see Table 1). However, the table also shows an increased risk of tumor recurrence compared with RUN, which is 21.9 and 13.1% respectively. Indeed, few studies reported outcomes of endoscopically managed UTUC with more than 50 months of follow-up. Cutress et al. reported a recurrence rate of 52 and 37% for ureteroscopic group and percutaneous nephroscopic group when reviewing 85 articles of endoscopic UTUC management [13]. It is notable that undergrading and missing of UTUC may result in severe consequences. In a study, up to 25% of patients had missed lesions, 50% of which had a missed carcinoma in situ lesion [14].

The evaluation of upper urinary tract evaluation is usually more intensive after endoscopic resection than after nephroureterectomy. However, follow-up after nephron-sparing management is difficult and it’s necessary to repeat endoscopic procedures. CT scanning cannot readily detect small tumors, thereby being unable to identify multifocal lesions. Ureteroscopic examination is regarded as a suitable diagnostic modality for detecting UTUC when other studies are ambiguous because it can offer direct visualization of the upper urinary tract. Moreover, it can obtain biopsy specimens during the procedure and confirm the tumor histology and grade [15–17]. Our patients were examined by ureteroscopy at 3-month intervals during the first year and at 6-month intervals since the second year. High-grade tumor cases are more likely to have recurrence as reported [18]. Four patients in our study developed ureteral stricture, all of which were treated with stenting, balloon dilatation, or laser incision successfully. Therefore, an intensive endoscopic surveillance is necessary during the follow-up.

This study has some limitations. Firstly, it is a retrospective study design and selection bias is inevitable between the two groups. Secondly, the number of patients is small and the follow-up time is short. Thirdly, some patients were treated by nephroureterectomy even after many years of follow-up because of progressive of disease. Nevertheless, we believe that our experience may contribute to the growing evidence.

## Conclusions

Thulium laser group is associated with a less loss of renal function, a shorter length of hospitalization, but a higher rate of tumor recurrence compared with nephroureterectomy group. Endoscopic treatment is feasible and safe for small, localized, low-grade and superficial UTUC under an intensive surveillance program.

## Abbreviations

BPH: Benign prostatic hyperplasia
ETLT: Endoscopic thulium laser treatment
RNU: Nephroureterectomy
UTUC: Upper tract urothelial carcinoma

## References

[1] Golan S, Nadu A, Lifshitz D (2015). The role of diagnostic ureteroscopy in the era of computed tomography urography. BMC Urol.

[2] Cho SY (2015). Current status of flexible ureteroscopy in urology. Korean J Urol.

[3] Oelke M, Bachmann A, Descazeaud A (2013). EAU guidelines on the treatment and follow-up of non-neurogenic male lower urinary tract symptoms including benign prostatic obstruction. Eur Urol.

[4] Muto G, Collura D, Giacobbe A (2014). Thulium: yttrium-aluminum-garnet laser for en bloc resection of bladder cancer: clinical and histopathologic advantages. Urol.

[5] Suriano F, Brancato T (2014). Nephron-sparing Management of upper tract urothelial carcinoma. Rev Urol.

[6] Verges DP, Lallas CD, Hubosky SG (2017). Endoscopic treatment of upper tract urothelial carcinoma. Curr Urol Rep.

[7] Roupret M, Zigeuner R, Palou J (2011). European guidelines for the diagnosis and management of upper urinary tract urothelial cell carcinomas: 2011 update. Eur Urol.

[8] Emiliani E, Herrmann TR, Breda A (2015). Thulium laser for the treatment of upper urinary tract carcinoma (UTUC), are we there, yet?. Word J Urol.

[9] Defidio L, De Dominicis M, Di Gianfrancesco L (2011). First collaborative experience with thulium laser ablation of localized upper urinary tract urothelial tumors retrograde intra-renal surgery. Arch Ital Urol Androl.

[10] Akopyan GN, Alyaev YG, Vinarov AZ (2016). Endoscopic removal of papillary tumors of upper urinary tract. Urologiia.

[11] Villa L, Cloutier J, Cotè JF (2016). Confocal laser Endomicroscopy in the Management of Endoscopically Treated Upper Urinary Tract Transitional Cell Carcinoma: preliminary data. J Endourol.

[12] Fried NM, Murray KE (2005). High-power thulium fiber laser ablation of urinary tissues at 1.94 microm. J Endourol.

[13] Cutress ML, Stewart GD, Zakikhani P (2012). Ureteroscopic and percutaneous management of upper tract urothelial carcinoma (UTUC): systematic review. BJU Int.

[14] Yamany T, van Batavia J, Ahn J (2015). Uretero¬renoscopy for upper tract urothelial carcinoma: how often are we missing lesions?. Urology.

[15] Roupret M, Babjuk M, Comperat E (2015). European Association of Urology guidelines on upper urinary tract urothelial cell carcinoma: 2015 update. Eur Urol.

[16] Rojas CP, Castle SM, Llanos CA (2013). Low biopsy volume in ureteroscopy does not affect tumor biopsy grading in upper tract urothelial carcinoma. Urol Oncol.

[17] Baard J, Freund JE, de la Rosette JJ (2017). New technologies for upper tract urothelial carcinoma management. Curr Opin Urol.

[18] Yamada Y, Inoue Y, Nakamura K (2009). Long-term results and management of ureteral transitional cell carcinoma using the holmium: YAG laser via rigid-ureteroscopy. Oncol Rep.

